# The effect of bone allografts combined with bone marrow stromal cells on the healing of segmental bone defects in a sheep model

**DOI:** 10.1186/1746-6148-10-36

**Published:** 2014-02-05

**Authors:** Marco Bernardo C Fernandes, João Antônio Matheus Guimarães, Priscila Ladeira Casado, Amanda dos Santos Cavalcanti, Natalia N Gonçalves, Carlos E Ambrósio, Fernando Rodrigues, Ana Carolina F Pinto, Maria Angélica Miglino, Maria Eugênia L Duarte

**Affiliations:** 1 Basic and Clinical Research Division, National Institute of Traumatology and Orthopedics, Rio de Janeiro, Brazil; 2 Institute of Biomedical Sciences, Federal University of Rio de Janeiro, Rio de Janeiro, Brazil; 3 Department of Veterinary Medicine, FZEA, USP. Faculdade de Zootecnia e Engenharia de Alimentos, Departamento de Medicina Veterinária, Universidade de São Paulo, Av. Duque de Caxias Norte, 225, 13635-900 Pirassununga, São Paulo, Brazil; 4 Department of Surgery, College of Veterinary Medicine and Animal Science, University of São Paulo, São Paulo, Brazil

**Keywords:** Allografts, Segmental bone defect, Bone marrow stromal cell, Animal model

## Abstract

**Background:**

The repair of large bone defects is a major orthopedic challenge because autologous bone grafts are not available in large amounts and because harvesting is often associated with donor-site morbidity. Considering that bone marrow stromal cells (BMSC) are responsible for the maintenance of bone turnover throughout life, we investigated bone repair at a site of a critically sized segmental defect in sheep tibia treated with BMSCs loaded onto allografts. The defect was created in the mid-portion of the tibial diaphysis of eight adult sheep, and the sheep were treated with *ex-vivo* expanded autologous BMSCs isolated from marrow aspirates and loaded onto cortical allografts (n = 4). The treated sheep were compared with control sheep that had been treated with cell-free allografts (n = 4) obtained from donors of the same breed as the receptor sheep.

**Results:**

The healing response was monitored by radiographs monthly and by computed tomography and histology at six, ten, fourteen, and eighteen weeks after surgery. For the cell-loaded allografts, union was established more rapidly at the interface between the host bone and the allograft, and the healing process was more conspicuous. Remodeling of the allograft was complete at 18 weeks in the cell-treated animals. Histologically, the marrow cavity was reestablished, with intertrabecular spaces being filled with adipose marrow and with evidence of focal hematopoiesis.

**Conclusions:**

Allografts cellularized with AOCs (allografts of osteoprogenitor cells) can generate great clinical outcomes to noncellularized allografts to consolidate, reshape, structurally and morphologically reconstruct bone and bone marrow in a relatively short period of time. These features make this strategy very attractive for clinical use in orthopedic bioengineering.

## Background

Since the beginning of the last century, several reports have generated interest in orthopedics regarding the use of allografts for the treatment of lost or damaged bone. The incidence of fracture, non-union and resorption of allografts is still of great concern because each has an occurrence rate as high as 20%, 17% and 11%, respectively Though the use of allografts is well known and accepted for the treatment of large bone defects, it still has a great amount of complications [1].

It is estimated that more than 1.5 million bone graft operations, in human, will be performed in the United States to enhance the healing of spinal fusion, for the internal fixation of fractures, for maxillofacial reconstruction, and for bone restoration at sites of bone loss due to trauma, osteolysis or ablative surgery [2-4].

Autologous bone grafts have osteogenic, osteoconductive, and osteoinductive properties. They have an excellent success rate, low risk of disease transmission, and high histocompatibility [5]. However, for many patients, autologous bone grafts are insufficiently osteogenic, available in limited supply, and associated with additional surgical morbidity, such as blood loss, nerve and arterial injury, and pain at the donor site. The disadvantages of bone allografts include their reduced osteoinductive capacity and their limited capacity to incorporate with the host bone [1,5,6].

Studies must be performed on the use of allografts with ceramic biomaterial loading of bone marrow stromal cells to treat similar large bone defects. These studies must show that the correct microenvironment, i.e., oxygen and nutrient, is generated and supplied to support the quality of the reconstructed scaffold [7].

Due to the limited ability to replace lost or diseased bone, living functional tissue engineering technologies are being developed to try to incorporate autologous bone graft properties, such as osteogenesis, into bone allografts.

The aim of the present study was to verify whether cultured autologous bone marrow stromal cells (BMSCs) loaded onto irradiated bone allografts would result in effective bone repair at the site of a large segmental defect in sheep tibia.

## Results

All animals experienced good postoperative recoveries: their surgical wounds healed well, and their microbiological tests showed no infection, because the implants were sterilized process by irradiated for sterilization. The cell samples from marrow aspirates (20–35 mL collected for each animal), for AOC 10 + and 14 + (animals with graft cellularized, ten and fourteen weeks before surgery), were obtained from the first pass, and for AOC 6 + and 18 + (animals with graft cellularized, six and eighteen weeks before surgery), were obtained from the second pass. In all cultures, regardless of the time of expansion, the cells had fusiform morphology characteristics of myofibroblastic cells. A similar process of culture isolation of bone marrow-derived mesenchymal stem cells was reviewed later and showed osteogenic and chondrogenic differentiation [8], which was different from the experimental cells.

**Figure 1:**
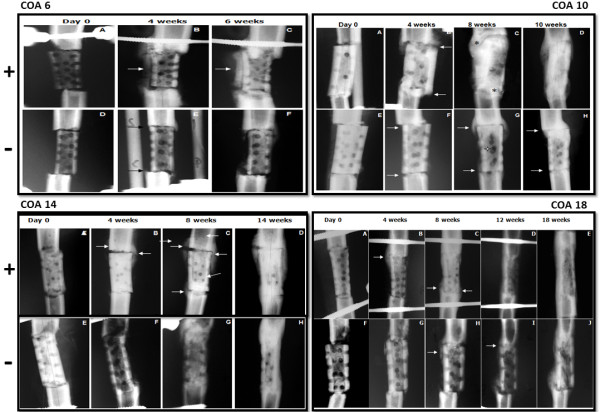
**Comparative radiographs of AOC 6 +, 10 +, 14 + and 18 + sheep and AOC 6 -, 10 -, 14 - and 18 - sheep.** Figures **A** and **D** (6), **A** and **E** (10), A and **E** (14) and **A** and **F** (18) illustrate the bone defect filled by a homologous bone graft over the weeks of the experiment.

**Figure 2:**
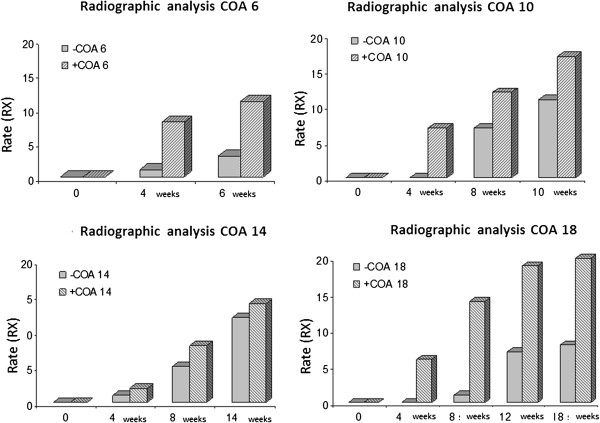
Graphic representation of the radiographic parameter scores for the AOC 6, 10, 14 and 18 animals in the evaluation points of 0, 4, 6, 10, 14, and 18 weeks.

The radiographic evolution of the grafts consolidation in the AOC 6+, 10 +, 14 +, and 18 + animals and the AOC 6 -, 10 -, 14 - and 18 - animals are illustrated in Figure 1. The parameters of the radiographic score of the two animals euthanized at 0, 4, 6, 10, 14 and 18 weeks are illustrated in Figure 2.

In both sheep, the grafts were aligned with the distal and proximal fragments (Figure 1A and D). After 4 weeks, the radiotransparent lines were less clear in the AOC 6 + animals because they were partially filled by new bone formation (Figure 1B). Bone formation in this region of the graft was significantly higher in the AOC 6- animals (Figure 1C) than in the animals treated with noncellularized grafts (Figure 1F).

Two months after surgery, in the AOC 10 + animals (Figure 1C), the graft was completely covered by new bone formation on the entire lateral surface. The osteotomy line with the host bone was practically imperceptible in most joints of the graft, indicating radiographic bone healing. The perforations of the cortical graft were partially filled by new bone formation, leaving few unconcluded holes. In the same period, for the AOC 10 - sheep (Figure 1G), the holes in the cortical graft and bone formation around the graft remained patent, although osteotomy was not significant and no callus formed a bone bridge between the graft and the host bone. The radiotransparent line remained visible in both outbreaks of osteotomy.

Two months after surgery, the AOC 14 + sheep (Figure 1C) showed large bone formations involving the lateral and medial osteotomy of the graft. Although the osteotomy lines were still evident, there was a formation of bony bridges joining the fragments in the proximal and distal focus. In the AOC 14 - sheep (Figure 1G), the bone formation was small and limited to the lateral focus of the proximal graft, but the osteotomy line was difficult to see due to the new bone formation.

In the AOC 18 + sheep, after three months (Figure 1D) the graft was completely consolidated and remodeled: it was no longer possible to identify the focus of the osteotomy. In the AOC 18 - sheep (Figure 1I), the osteotomy focus was consolidated proximally and distally and remained fully visible, indicating delayed consolidation. At 18 weeks, the cellularized grafts (Figure 1E) showed an advanced process of remodeling, with the thickening of the cortical region and partial reduction of the thickness of the callus formed at the foci of the osteotomy. For sheep in the control group (Figure 1J), although the graft was consolidated at both foci of the osteotomy, it was still possible to visualize the radiotransparent line in distal focus.

At the first analysis point (6 weeks), perforations were clearly identified in the cortical grafts, and no difference was observed between the two animals with regards to the filling of the holes by new bone formation. However, as in the radiographic evaluation, the formation of the callus and the periosteal reaction were more evident in the animals with cellularized grafts (Figure 2A and B, AOC 6 + and 6 -). At 10 and 14 weeks, the animals treated with cellularized grafts (AOC 10 + and 14 +) had holes that were less patent than the control animals (AOC 10 - and AOC 14 -) due to increased bone formation and, consequently, greater obliteration of the perforations (Figure 2A, B, C and D).

**Figure 3:**
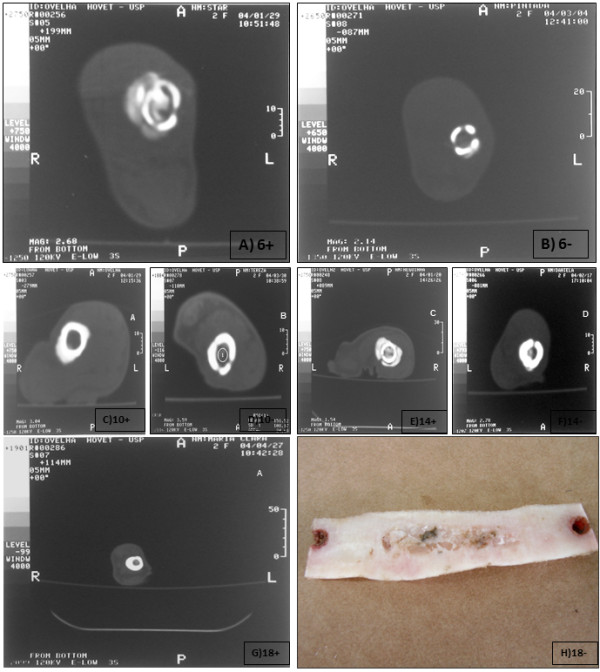
**Tomography analyses during bone healing, after stem cell therapy, performed in the medial plane of the grafts of animals euthanized at 0, 6, 10, 14 and 18 weeks after the correction of diaphyseal segmental defects with cellularized homologous (A) and noncellularized homologous (B) grafts.** The holes corresponding to the holes in the compact cortical bone are apparent in both animals. (Figure 3**A** to 3**G**). Gross piece of bone corresponding to the macroscopic analyses of the AOC 18 + animal (3**H**).

After 18 weeks, the perforations of the cortical bone of the recipient cellularized grafts (AOC 18+) were completely filled by new bone formation and were no longer displaying tomographic sections (Figure 3A to 3G following complete healing of the bone and comparative disappearance of holes dependent of therapy and 3H as gross cross section of new formed bone structure, AOC 18 + and 18 -).

Histopathological evaluation was performed on the entire length of the graft, including the interface with the host bone. To standardize the results and thus facilitate the comparative analysis of the bone repair process in the presence and absence of AOC, the histological findings were correlated between the animals euthanized at each of the four observation points.

**Figure 4:**
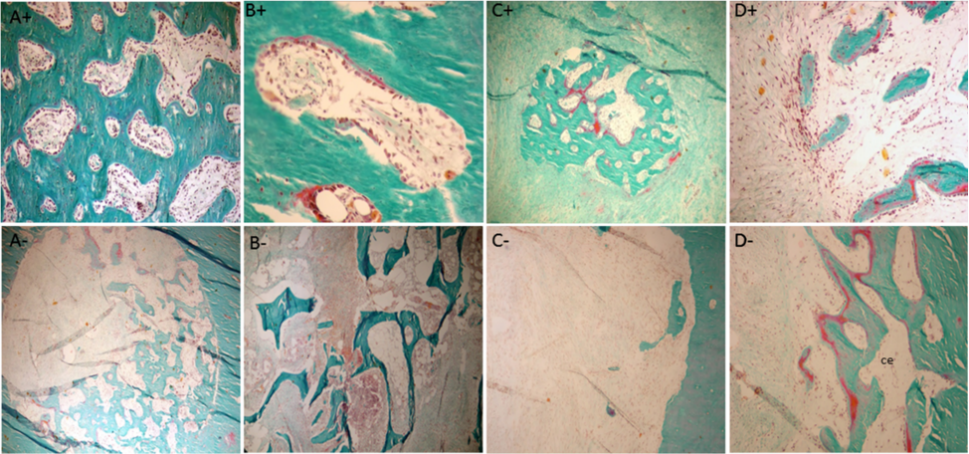
**Histological appearance of the graft in AOC 6 - sheep. (A)** Orifice corresponding to the perforation of the cortical graft only partially filled with new bone formation. **(B)** Intact allogeneic bone graft represents most of the filling of the inner portion of the graft. **(C)** Portions of the filling of the graft are represented mostly by connective tissue with no evidence of active osteogenesis. **(D)** Osteogenesis, when identified in the inner portion of the implant, is represented by partially mineralized bone trabeculae (osteoid seams stained in red). Masson-Goldner trichrome stain; 5-mm-thick undecalcified sections. Magnification **(A-D)** - 4×. Histological appearance of the graft in AOC 6 + sheep. **(A)** The site of the perforation in the cortical graft is filled by well-connected thick bone trabeculae displaying high osteoblastic activity through practically the entire length of the trabecular surfaces. **(B)** Cortical graft undergoing remodeling (*cutting cone*). **(C)** Focal area of active osteogenesis localized in the connective tissue that fills the central portion of the graft. **(D)** Bone formation in close association with loose connective tissue featuring characteristics of bone marrow supporting stroma. Masson-Goldner trichrome stain; 5-μm-thick undecalcified sections. Magnification 10× **(A and B)**, 4× **(C and D).**

After 6 weeks, in the AOC 6 + animal, the holes of the compact bone graft were filled and consisted largely of bone formation and structured thick beams interconnected with marked osteoblastic activity. In the peripheral portion of the hole, the neoformation of immature bone tissue predominated and was related with high osteogenic cell activity. In the compact bone graft, there were frequent outbreaks of osteoclastic resorption of both the Haversian and cortico-endosteal surface. Haversian resorption occurred in an organized manner, and the presence of cutting-cones was characteristic of bone remodeling. The main components of the medullary filling at this vantage point were tissues and beams of neoformed bone in similar proportions. The peri-trabecular tissue in the general aspect was loose and highly vascularized, morphologically similar to the bone marrow stroma (Figure 4A^+^ to D^+^).

For the AOC 6 - animal, interfaces with the host bone graft were filled by dense connective tissue and bone formation inside the hole penetrated into the medullary cavity. However, the main difference observed by the comparison of the AOC 6 - animal with the AOC 6 + animal was the composition of the tissue filling the medullary space (Figure 4A^-^ to D^-^).

**Figure 5:**
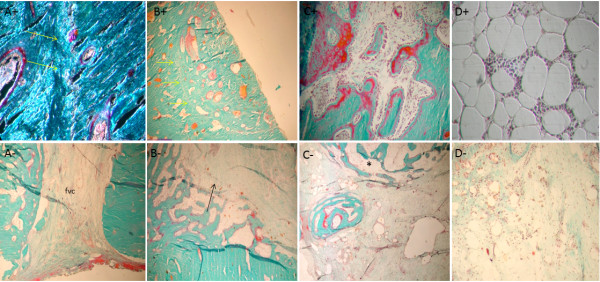
**Histological appearance of the grafts in AOC 10 + sheep. (A)** Line of fusion (arrows) in the interface between the distal portion of the cortical graft (right) and the host bone (left). **(B)** Corresponding site of a perforation of the cortical graft completely filled by well-organized bone formation. Arrows indicate the boundary of the former orifice. **(C)** Osteogenesis occurring in the inner portion of the graft. Bone trabeculae are covered by osteoid seams in close relationship with numerous active plump osteoblasts. **(D)** Adult adipocytes and classical hematopoietic cells are observed within the graft in the restored bone marrow. Masson-Goldner trichrome stain; 5- μm -thick undecalcified sections. Magnification 10× (**A**, under polarized light and **C**), **(B)** 4×, **(D)** 20×. Histological appearance of the graft in AOC 10 - sheep. **(A)** Gap between host bone and allograft containing fibrous connective tissue with no evidence of osteogenesis. **(B)** Interface between host bone and allograft displaying bone formation towards the inner portion of the medullary cavity (arrow). **(C)** The connective tissue that partially fills the central portion of the graft displaying a focal area of active osteogenesis intimately related with loose connective tissue, with the morphology of bone marrow supporting stroma (*). **(D)** Remainder of cancellous graft being actively resorbed by osteoclasts, without evidence of active bone formation. Masson-Goldner trichrome stain; 5- μm -thick undecalcified sections. Magnification **(A-D)** - 4×.

After 10 weeks of observation, the animals treated with cellularized grafts (AOC 10 +) had bone continuity in the region of the distal osteotomy. The compact bone graft was in the process of remodeling, and morphological structures of osteons were often identified. Although much of the graft that filled the medullary cavity consisted of bone formation, this was not uniform, and the beams filling the cavity were not structured because the interconnected distal edge of the graft and the host bone were joined by trabecular bone (Figure 5A^+^ to D^+^).

In the AOC 10 + animal, most of the tissue present in the marrow cavity of the graft was replaced by new bone. In the animal treated with a noncellularized graft (AOC 10 - ), extensive areas of connective tissue and fbro-vascular tissue was observed and just a little or no osteogenic activity, differently from results found for AOC 10+ (Figure 5A^-^ to D^-^).

**Figure 6:**
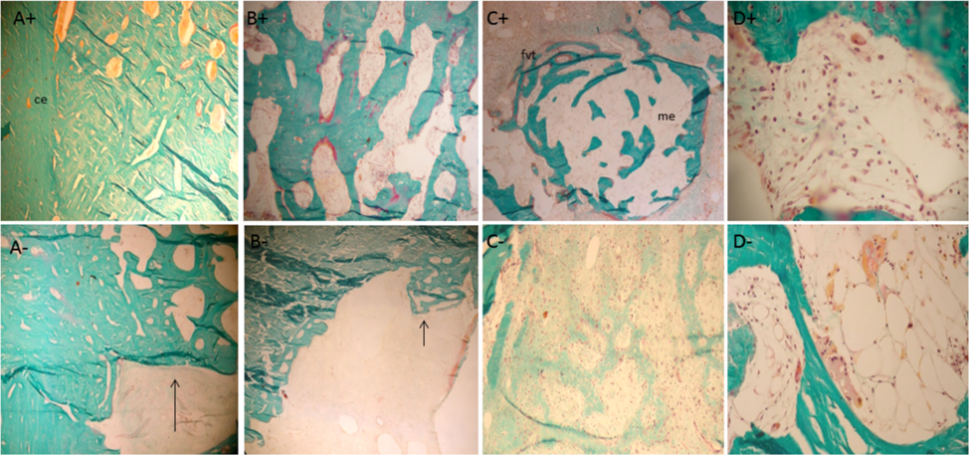
**Histological appearance of the graft in AOC 14 + sheep. (A)** Solid union between the allograft and the host bone depicted by well-organized and well-connected thick bone trabeculae. **(B)** Inner portion of the allograft filled mostly by mature bone trabeculae with sites of active new bone deposition represented by the presence of osteoid seams stained in red and covering portions of the trabecular surface. **(C)** Osteogenesis in the connective tissue that fills the allograft. Note the similarity of the loose connective tissue associated with the newly formed bone trabeculae and the supporting stroma of normal bone marrow. This aspect is histologically distinct from the clearly non-osteogenic fibro-vascular connective tissue that surrounds the site of osteogenesis. **(D)** Restoration of the bone marrow microenvironment with the colonization of the supporting marrow stroma by hematopoietic cells. Masson-Goldner trichrome stain; 5- μm -thick undecalcified sections. Magnification **(A-C)** 4× and **(D)** 20×. Histological appearance of the graft in AOC 14 - sheep. **(A)** Despite the osseous union achieved between the host bone and the allograft, the inner portion of the graft is devoid of osseous filling. **(B)** Inner portion of the cortical graft occupied by connective tissue with little or no osteogenic potential. **(C)** Most of the osteoforming response in this graft was represented by woven bone in an initial state of formation. **(D)** Remnants of cancellous graft are being actively resorbed by multinucleated osteoclasts without signs of restoration of the bone marrow microenvironment. Masson-Goldner trichrome stain; 5- μm -thick undecalcified sections. Magnification **(A-C)** 4× and **(D)** 10×.

In the AOC 14 + animal, both interfaces of the graft were filled by newly formed bone, which established the union between the grafted bone and native bone. In the AOC 14 - animal, the medullary filling was not consistent and differed depending on the proximity to the edges of the host bone. In the regions immediately adjacent to the interfaces of the graft, the marrow space was filled mostly by dense connective tissue containing empty cavities and newly formed vessels (Figure 6A^+^ to D^+^ and A^-^ to D^-^).

**Figure 7:**
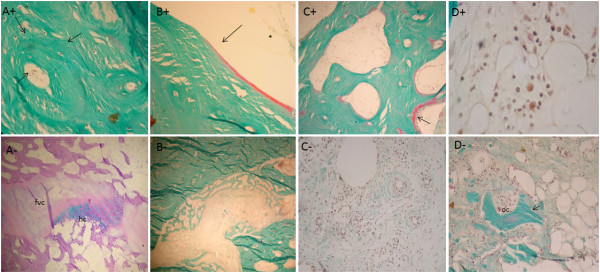
**Histological appearance of the graft in AOC 18 + sheep. (A)** Complete remodeling of cortical graft resulted in well-organized cortical bone with osteons displaying the classical concentric arrangement of the bone lamellae in close association with osteocytes. **(B)** Cortico-endosteal surface in a resting state, covered by a thin inactive osteoid seam (arrow) indicating that the remodeling process at this site was accomplished. **(C)** Structural restoration of the marrow cavity is evidenced by the presence of well-organized mature bone trabeculae undergoing remodeling (arrow). **(D)** The restoration of the bone marrow microenvironment can be confirmed by the presence of hematopoietic cells. Masson-Goldner trichrome stain; 5- μm -thick undecalcified sections. Magnification **(A-C)** 4× and **(D)** 20×. Histological appearance of the graft in AOC 18 - sheep. **(A)** Although an osseous union was accomplished at both interfaces with the host bone in CMA 18 + sheep, in CMA 18 - sheep, cartilage (ha) remnants and connective tissue (fvc) were still present at this location. This finding characterizes the incomplete incorporation of the graft. **(B)** Orifice corresponding to the perforation of the cortical graft incompletely filled with bone, suggesting that bone formation is not yet complete. **(C)** Despite the formation of highly vascular connective tissue in the inner portion the graft, there is no evidence of osteogenesis. **(D)** Compared with CMA 18 + sheep, the delay in the process of consolidation and remodeling of the allograft can be exemplified by the presence of residual cancellous graft (arrow) undergoing osteoclastic resorption (oc). Masson-Goldner trichrome stain; 5- μm -thick undecalcified sections. Magnification **(A and B)** 4×, **(C and D)** 10×.

After 18 weeks, in the animals treated with cellularized grafts (AOC 18 +), the regions of the graft interfaces were no longer histologically identifiable. The compact bone graft in this region comprised morphological structures with deposits of osteons, concentric lamellar bone osteocytes and round vascular structures. With the completion of the process of consolidation and remodeling of the callus, the endosteal and periosteal surfaces showed no more cellular activity, lying covered with thin ledge osteoid unrelated osteoblasts that featured inactive or latent state bone surfaces (Figure 7A^+^ to D^+^).

The comparison between the two animals euthanized after 18 weeks showed significant differences in the pattern and temporal evolution of the bone healing process at the graft site. Unlike what was observed in AOC 18 + animals, where the remodeling of the bone callus had been completed, the AOC 18 – animal (Figure 7A^-^ to D^-^) exhibited a periosteal reaction covering a large part of the graft, including the region of the osteotomies. This finding confirmed that the process of graft consolidation was still in progress.

Inside the graft tissue, there was no uniformity in the filling composition. In the central portion, the tissue was represented by beams of bone formed from the cortico-endosteal surface and the intramedullary tissue and by fibrovascular tissue unattached to osteogenesis. The filling in the less central tissue alternated between beams with thick bone cell activity foci, a good standard of connectivity and related connective tissue stroma with features of bone marrow fat and areas represented exclusively by intensely vascularized tissue, unrelated to training of bone.

## Discussion

The results of this study demonstrate that large diaphyseal defects can be effectively treated with irradiated homologous grafts in combination with external fixation and autologous osteoprogenitor cells, which, when added to these grafts, accelerate bone formation and the incorporation and remodeling of the graft in segmental diaphyseal defects and do not trigger local or systemic adverse reactions. The results also showed that the addition of the precipitating cells of the bone graft substituted for cancellous bone, cortical bone and bone marrow. In this study, in an experimental mammalian model, the treatment of allografts augmented with bone marrow stromal cells proved to be an effective option for ameliorating bone loss extending into the tibial shaft.

The results of pre-clinical studies [9-13] and clinical trials [14,15] using autologous mesenchymal cells and osteoprogenitors isolated from bone marrow and expanded in vitro to treat hydroxyapatite associated with diaphyseal defects were highly favorable. The recovery of the injuries occurred through the complete repair of the bone defects, with no history of local or systemic complications directly related to the implant or cellular components.

The alternative use of adipose tissue to isolate the osteoprogenitor cells may lead to insufficient bone formation, as observed in a previous study [16]. It is fairly well established that the growth of pluripotent cells isolated from adipose tissue generate a large number of progenitors committed to the adipogenic lineage with little or no ability to trans-differentiate into osteogenic cells [16].

Much of the observations of the pioneer study [17] are in agreement with our results. The authors of the pioneer study filled bone defects with a cylinder of hydroxyapatite and tri-calcium phosphate cellularized with mesenchymal cells that were expanded in vitro. Some disadvantages and complications of using this type of synthetic material, such as slow absorption and the occurrence of fractures, were infrequent in animals treated with cellularized grafts. As in our study, the most significant result from the histological point of view was the ability of cells added to the graft to induce bone formation.

As suggested in a previous study [18], mesenchymal cells that are in the process of osteogenic differentiation secrete factors that induce the recruitment of new cells and mesenchymal differentiation in the osteogenic lineage. The exuberance of the periosteal reaction in the host animals treated with cellularized bone grafts could be explained, at least in part, by the paracrine effects of these factors synthesized by the implanted cells. Pluripotent mesenchymal cells, in both the periosteum and the bone, respond to these factors, differentiating into osteoblasts and mature bone matrix producing cells. This hypothesis also applies to the slower bone formation and reduction of defects in the animals treated with noncellularized grafts. Recent studies complement this hypothesis [19] by demonstrating that TGF, BMP, FGF, IGF and PDGF are the main growth factors able to influence the proliferation and differentiation of mesenchymal cells into osteoprogenitors and osteoblasts [20].

There are other case studies on bone grafts in sheep that deserve special attention [20-23]. Furthermore, a clinical study on the use of mesenchymal cells expanded in vitro and associated with a synthetic implant was performed in four patients with segmental defects [24]. Monitoring these patients for 6–7 years after surgery showed that there were no early or late complications. There were no reports of pain, swelling, infection or fracture. The integration of the implant observed after 5–7 months was maintained 6–7 years after surgery. Though the results were favorable, despite the low degree of resorption and mechanical stability of the hydroxyapatite compounds, we suggest that superior clinical outcomes can be achieved through the association of allografts of osteoprogenitor cells as autologous cells.

## Conclusions

Allografts cellularized with AOCs (allografts of osteoprogenitor cells) can generate great clinical outcomes to noncellularized allografts to consolidate, reshape, structurally and morphologically reconstruct bone and bone marrow in a relatively short period of time. These features make this strategy very attractive for clinical use in orthopedic bioengineering and open new venue for veterinary medicine bone surgery.

## Methods

### Experimental design

Ten (eight formed the experimental and control group and 2 animals is donors) adult female sheep (26–28 months) weighing 36 ± 2.7 kg were used in the present study after approval of the Ethical Committee for Animal Research at The University of São Paulo (authorization 317/2003). In all the animals, a central 30 mm bone segment was resected from the mid-portion of the right tibial diaphysis. The defect was replaced with a cylindrical graft of homologous cortical bone containing internal fragments of spongy bone. In four animals, osteoprogenitor cells were added to the autologous bone grafts (AOC 4+, n = 4); control animals were treated with cell-free allografts (AOC 4-, n = 4). Before the beginning of the experiment, two animals of the same age, sex and breed from the group of experimental animals were euthanized with an overdose of thiopentone and were used as bone (long bones mainly) allograft donors.

All animals were monitored with radiographs in the immediate postoperative period and monthly thereafter. Six, ten, fourteen, and eighteen weeks (AOC 6+/−, 10+/−, 14+/ 18+/−) after surgery, the animals were euthanized, and after removal of the external fixator, their right hind limb was evaluated by computed tomography. After the completion of CT and plain radiographs, the right tibia, which contained the bone graft, was resected and subsequently processed for histological analysis.

### Preparation of the allograft

After skeletonization, 30 mm cortical cylinders were obtained from the long bones of two donor sheep of the same breed, sex and age as the receptor sheep. Under sterile conditions, a central canal was bored through the length of the entire cylinder with the use of an 8 mm electrical drill. Cortical allografts were further perforated with a 3.2 mm high-speed drill to form equidistant holes along the entire surface, with approximately 4 mm between each hole. All bone grafts used, consisting of approximately 10×5 mm bone fragments were obtained from iliac crests and metaphyses of the long bones of the donor sheep. Both types of allografts were washed throughout this process with saline and were then placed for 10 minutes in a saline solution containing 0.02 g/ml of cephalotin and stored individually in plastic bags. Both types of allografts were kept frozen at −70°C, including during the sterilization process by γ-radiations (25,000 gray). Immediately before use, the samples were thawed in sterile saline.

### Surgical procedure

**Figure 8:**
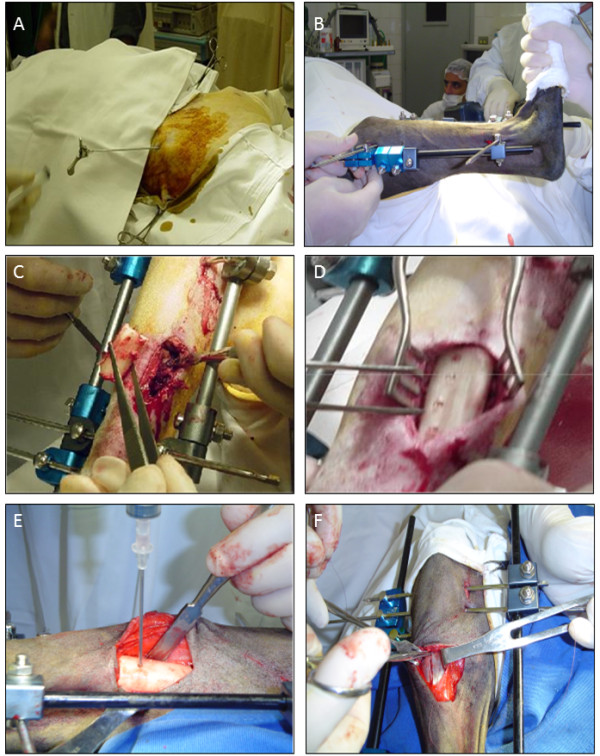
**Procedures for bone marrow collection and surgical techniques for segmental defects establishment and stem cell transplantation. A)** Puncture procedure of the left iliac crest for obtaining bone marrow aspirate. **B)** Monoplanar assembly positioned bilateral to external fixator. **C)** Creation of a 3.5 cm segmental defect in the middle third of the right tibial shaft. **D)** Cells were injected through the holes in the cortical graft to fill the bone defect. **E)** Cell suspension injection through the holes in the cortical graft. **F)** Suture of the incision of the periosteum at the end of the surgical procedure to prevent overflow of cell suspension.

Under general anesthesia and using endotracheal intubation, the right hind limb was shaved, and the area over the tibia was aseptically prepared. Mechanical stability was achieved with static external fixation (Baumer, São Paulo, Brazil) using a bilateral monoplanar frame with six parallel pins inserted from the lateral to the medial side of the tibia. With the animal placed on its left side, a 30 mm diaphyseal segment between the proximal and distal groups of pins was dissected subperiosteally, osteotomized and explanted, creating a segmental bone defect. The defect was replaced with a cortical sterile allograft and filled with scaffolds previously harvested from the donor sheep. In the experimental animals, autologous expanded BMSCs were subsequently loaded onto the scaffolds by injecting 2 mL of the cell solution with a 5 mL syringe through the 3.2 mm screw holes. Unrestricted weight bearing was allowed for all animals immediately after the operation (Figure 8A-F). Non-steroidal anti-inflammatory drugs were given for two days postoperatively, and after a week in isolated cages, the sheep returned to regular housing facilities, where they were monitored daily. The animals were euthanized by an intravenous injection of potassium chloride under sodium pentobarbital anesthesia 6, 10, 14 and 18 weeks after surgery.

### Anesthesia and post-surgical procedures

After 24 hours of no intake food and 12 of not drinking water, animals were pre-medicated with midazolam maleate (0.6 mg/kg) and meperidine hydrochloride (2 mg/kg) intravenously (IV). Anesthesia was induced by propophol (3 mg/kg) associated to fentanyl hydrochloride (2.5 μg/kg) and maintained with isoflurorane. Post-surgical analgesia was provided with 1 mg/kg of flunixin meglumine and 25 mg/kg of sodium dipyrone IV and maintained with 2 mg/kg of tramadol twice a day until 10 days. Antibacterial prophylaxis was obtained with penicillin 40000 UI/kg for 10 days [25].

### Isolation and culture of sheep BMSCs

20–30 mL of bone marrow aspirates was harvested from the iliac crest of the experimental sheep. The animals were anesthetized, and under sterile conditions, a 15-gauge trephine was used to aspirate the bone marrow into a 10 mL syringe rinsed with 1000 UI/mL heparin. Marrow samples were diluted into Dulbecco’s modified Eagle’s medium (DMEM) and filtered through a 150 μm cell strainer. Nucleated cells were plated at a density of 6×107 in DMEM supplemented with 10% fetal bovine serum (FBS) and 1% antibiotics in 150-cm^2^ flasks at 37°C in a humidified atmosphere of 5% CO_2_. After 24 hours, non-adherent cells were washed out, and adherent cells were then fed with fresh medium twice a week. When the cells were nearly confluent, they were harvested with 0.05% trypsin/0.01% EDTA, counted and seeded at a density of 6×107 in 150 cm^2^ tissue-culture flasks.

Autologous BMSCs were expanded until passage 2 and suspended in 2.0 mL of implantation media (DMEM containing 10% FBS). From the time of preparation until the implantation procedure (4–5 hours), the cell suspension was kept at 4°C. In the operating room and immediately before use, the cell suspension was submitted to gentle mechanical homogenization, aspirated in 5 mL syringes and introduced into the positioned allograft through the cortical perforations.

### Radiographic and tomographic analysis

All animals were regularly monitored radiographically after surgery and every month during the experimental period. The healing process was evaluated by antero-posterior and lateral radiographs and by computed tomography after *post-mortem* removal of the external fixator. Two observers (M. B. C. F and M. E. L. D) independently and blindly evaluated the presence of the periosteal reaction, host-graft union and the general graft appearance, which included partial absorption and graft remodeling according to a previously established scoring system (Table 1).

**Table 1:** **Radiographic criteria for evaluating the consolidation of adapted bone grafts**[19]

	**Points**	
A. Periosteal reaction	Forelimb	Hindlimb
Absent	0	0
Minimum	1	1
Medium (<50%)	2	2
Moderate (50-75%)	3	3
Complete (>75%)	4	4
B. Graft host bone union	Proximal limb	Distal limb
Radiotransparent line (total)	0	0
Radiotransparent line (partial)	2	2
Absent radiotransparent line	4	4
C. Graft changes		
Reaction absent	0	
Partial absorption	1	
Moderate remodeling	2	
Complete remodeling	3	
Total organization	4	
D. Complications (fracture or graft resorption)		
Total points	0	

### Histological analysis of retrieved allografts

The tibia was explanted at the time of euthanasia, and the middle part of the diaphysis, including the allograft and both host bone-implant interfaces, was excised. After removal of the surrounding soft tissues, tibial diaphysis specimens were obtained from the entire length of the allograft and from the osteotomy sites including the host bone with an oscillating saw. Macroscopic slices were fixed in 70% ethanol and dehydrated at 4°C by sequential changes in ascending concentrations of ethanol, cleared in xylene and embedded in methyl-methacrylate. Five-micron undecalcified bone sections were obtained using a Leica RM 2155 microtome (Leica, Nussloch, Germany) equipped with a disposable carbide-tungsten knife. Approximately 15–20 undecalcified histological sections taken from each animal were stained with Goldner´s Trichrome and PAS/Alcian Blue stain and examined with the use of a light microscope (Nikon Eclipse E800, Nikon Corporation, Japan). The reparative process in retrieved allografts was qualitatively assessed in both gaps at the osteotomy site, at the external and internal cortical surfaces of the grafted bone and internally in the medullary canal.

## Competing interests

The authors declare that they have no competing interests.

## Authors’ contributions

MBCF, MELD designs the experiment. With first MBCF and MELD, JAMG, PLC, ASC, NJG, CEA, FR, ACFP, MAM prepared the animals, performed the surgery and material analysis consisting radiographic, sectioned and stained the bones, reviewed the literature and prepared the manuscript. All authors read and approved the final manuscript.
